# Coexpression of EphB4 and ephrinB2 in tumour advancement of ovarian cancers

**DOI:** 10.1038/sj.bjc.6604216

**Published:** 2008-01-29

**Authors:** S M Alam, J Fujimoto, I Jahan, E Sato, T Tamaya

**Affiliations:** 1 Department of Obstetrics and Gynecology, Gifu University School of Medicine, Yanagido 1-1, Gifu City 501-1194, Japan

**Keywords:** EphB4, ephrinB2, prognostic indicator, tumour advancement, ovarian cancers

## Abstract

EphB4 and ephrinB2 expressions in ovarian cancers were studied to analyse EphB4/ephrinB2 functions against clinical backgrounds. EphB4 and ephrinB2 were dominantly localised in ovarian cancer cells of all cases studied. Both the histoscores and mRNA levels of EphB4 and ephrinB2 significantly increased with clinical stages (I<II<III<IV, *P*<0.001) in ovarian cancers, although there was no significant difference in EphB4 and ephrinB2 histoscores or in mRNA levels according to histopathological types. EphB4 as well as ephrinB2 histoscores in cancer cells correlated with the corresponding mRNA levels in each case (EphB4, *P*<0.001; ephrinB2, *P*<0.001). The 24-month survival rates of the 36 patients with high EphB4 and ephrinB2 expression were poor (25 and 27%, respectively), while for the other 36 patients with low EphB4 and ephrinB2 expression, they were significantly higher (68 and 64%, respectively). Therefore, EphB4/ephrinB2 may function in tumour advancement and coexpression of the Eph/ephrin system may potentiate tumour progression leading to poor survival. Thus, EphB4/ephrinB2 can be recognised as a novel prognostic indicator in the primary tumours of ovarian cancers.

Receptor tyrosine kinases (RTKs) are a diverse group of transmembrane proteins that, upon receiving an external stimulus, respond by transmitting a signal to the inside of the cell and thus control the cell shape, proliferation, differentiation and migration (Hunter *et al*, 1992; Dodelet and Pasquale, 2000; Lamorte and Park, 2001). In that sense, tyrosine kinase receptors have significant roles in normal physiology and in oncogenesis (Nakamoto and Bergemann, 2002). Of all the RTKs that are found in the human genome, the Eph receptors constitute the largest subfamily of RTK proteins and all members share a similar structure including a ligand binding extracellular domain, a single transmembrane domain and an intracellular tyrosine kinase domain. As a unique class of RTKs, the Eph family, first discovered in a human cDNA library screen for homologous sequences to the viral oncogene *vfps* (Hirai *et al*, 1987), consists of at least 14 receptors and 8 ligands (Gale *et al*, 1996; Eph Nomenclature Committee, 1997). Unlike other families of RTKs, which bind soluble ligands, Eph receptors interact with cell-surface-bound ephrin ligands. Based on binding preference and sequence homology, the Eph receptors have been divided into two subclasses, EphA (A1–A8) and EphB (B1–B6) (Gale *et al*, 1996). EphA receptors are anchored on plasma membranes through a glycosylphosphatidylinositol linkage and bound by their ligand ephrinA (A1–A5), whereas EphB receptors are bound by ephrinB (B1–B3) and tethered to the membrane by a transmembrane domain (Gale *et al*, 1996). EphA4 is an exception in that it can bind A type and most B type ligands.

EphB4 selectively binds ephrinB2 and no other ephrinB ligands (Brambilla *et al*, 1995; Sakano *et al*, 1996). EphrinB is constituted of transmembrane proteins with an intracellular domain that can elaborate reverse signalling (Davis *et al*, 1994; Gale and Yancopoulos, 1997). Ligand–receptor binding leads to protein clustering followed by receptor activation (Davis *et al*, 1994). Besides, both Ephs and ephrins are membrane bound and therefore binding and activation require cell-to-cell interaction rather than long-range communication; they mediate bidirectional signalling cascades (Fuller *et al*, 2003). When EphB4 is activated by ephrinB2, multiple tyrosine sites are phosphorylated and the kinase domains activated, although soluble forms of either ephrinA or ephrinB cannot activate their receptors (Davis *et al*, 1994; Katoh and Pasquale, 1999).

EphB4 plays an important role in a variety of processes during embryonic development, including pattern formation, cell aggregation and migration, segmentation, neural development, angiogenesis and vascular hierarchical remodelling (Pasquale, 1997; Gerety *et al*, 1999; Tickle and Altabef, 1999). EphrinB2 participates in vascular remodeling, maturation and directed growth (Tickle and Altabef, 1999). Coexpression of both EphB4 and ephrinB2 at high levels in malignancies including melanoma, neuroblastoma and cancers of the prostate, breast, lungs, oesophagus, gastrium, colorectum and uterine endometrium dysregulates cell adhesion and cell motility in tumours (Kyokawa *et al*, 1994; Vogt *et al*, 1998; Easty *et al*, 1999; Walker-Daniels *et al*, 1999; Tang *et al*, 2000; Miyazaki *et al*, 2003). To discover additional molecular therapeutic targets that may be incorporated into a multimodality regimen for the treatment of ovarian cancer, we studied the expression and localisation of the EphB4 receptor and the corresponding ephrinB2 ligand in ovarian cancers to analyse EphB4/ephrinB2 functions against clinical backgrounds.

## MATERIALS AND METHODS

### Patients and tissues

Prior informed consent for the following studies was obtained from all patients and the study was approved by the Research Committee for Human Subjects, Gifu University School of Medicine. Seventy-two patients ranging from 45 to 79 years of age with ovarian cancers (15 stage I cases, 19 stage II cases, 28 stage III cases and 10 stage IV cases and 13 cases of clear cell adenocarcinoma, 8 cases of endometrioid adenocarcinoma, 18 cases of mucinous cystadenocarcinoma, 15 cases of serous cystadenocarcinoma and 18 cases of serous papillary cystadenocarcinoma) underwent surgical resection, which produced macroscopically disease-free status, at the Department of Obstetrics and Gynecology, Gifu University School of Medicine, between September 1997 and March 2003. None of the patients had received any preoperative therapy. The tissues of ovarian cancer were obtained immediately after surgery. The tissues for RNA isolation were snap-frozen and stored at −80°C, and those for immunohistochemistry were fixed with 10% formalin and embedded in paraffin wax. The clinical backgrounds of ovarian cancer were evaluated by the International Federation of Gynecology and Obstetrics (FIGO) classification (FIGO News, 1989).

### Immunohistochemistry

Four-micrometre-thick sections of formalin-fixed paraffin-embedded tissue samples from ovarian cancers were cut with a microtome and dried overnight at 37°C on a silanised slide (Dako, Carpinteria, CA, USA). The protocol of universal Dako Labelled Streptavidin-Biotin kit (Dako) was followed for each sample. Samples were deparaffinised in xylene at room temperature for 30 min, rehydrated with graded ethanol and washed in phosphate-buffered saline (PBS). The samples were then placed in 10 mM citrate buffer (pH 6.0) and boiled in a microwave for 10 min for epitope retrieval. Endogenous peroxidase activity was quenched by incubating tissue sections in 3% H_2_O_2_ for 10 min. The primary antibodies, rabbit EphB4 (sc-5536, Santa Cruz Biotechnology, Santa Cruz, CA, USA) and rabbit ephrinB2 (sc-1010, Santa Cruz) were used overnight at 4°C at dilutions of 1 : 100 and 1 : 75, respectively. The slides were washed and biotinylated anti-rabbit secondary antibody (Dako) was applied for 30 min. After rinsing in PBS, streptavidin-conjugated horseradish peroxidase (Dako) was added for 30 min. Slides were then washed and treated with the chromogen 3,3′-diaminobenzidine (Dako) for 5 min, rinsed in PBS and counterstained with Mayers haematoxylin, dehydrated in graded ethanol, cleared in xylene and coverslipped with a mounting medium, Entellan New (Merck, Darmstadt, Germany). Rabbit preimmune animal serum (Dako) was used for negative controls instead of the primary antibody for EphB4 or ephrinB2.

### Assessment of histochemical score

All sections of immunohistochemical staining for EphB4 and ephrinB2 were evaluated in a semiquantitative fashion by two pathologists according to the method described by McCarty *et al* (1985), which considers both the intensity and the percentage of cells stained at each intensity. Intensities were classified as 0 (no staining), 1 (weak staining), 2 (distinct staining), 3 (strong staining) and 4 (very strong staining). For each stained section, a value-designated histochemical score (histoscore) was obtained using the algorithm, histoscore=∑(*i*+1)*Pi*, where *i* and *Pi* represent the intensity and percentage of cells that stain at each intensity, respectively, and the corresponding histoscores were calculated separately.

### Preparation of standard template for real-time reverse transcription–PCR

Internal standard template for real-time PCR was produced by PCR amplification using the primers for EphB4 gene, 2201–2682 in the cDNA (EphB4-TS: 5′-AGATCGATGTCTCCTACGTC-3′ and EphB4-TAS: 5′-GATTCCTGGAGGAGAACTCT-3′) and for ephrinB2 gene, 570–989 in the cDNA (ephrinB2-TS: 5′-GTCCAGAACTAGAAGCTGGT-3′ and ephrinB2-TAS: 5′-GTACATCGTCCAGGAGATGC-3′). The DNA template was purified using a GeneClean II kit (Qbiogene, Irvine, CA, USA). The copy numbers of the standard template were determined to quantify EphB4 and ephrinB2 mRNA levels in samples for real-time reverse transcription–PCR (RT–PCR).

### Real-time RT–PCR

Total RNA was extracted with the acid guanidinium thiocyanate–phenol–chloroform method (Chomczynski and Sacchi, 1987). The total RNA (3 *μ*g) was reverse transcribed using Moloney murine leukaemia virus reverse transcriptase (MMLV-RT, 200 U *μ*l^−1^; Invitrogen, Carlsbad, CA, USA) and the following reagents: 250 mM Tris-HCl, pH 8.3, 375 mM KCl, 15 mM MgCl_2_, 0.1 M dithiothreitol, 10 mM deoxynucleotide (deoxyadenosine, deoxythymidine, deoxyguanosine and deoxycytidine) triphosphates (dNTPs) mixture and random hexamers (Invitrogen) at 37°C for 1 h. The reaction mixture was heated for 5 min at 94°C to inactivate MMLV-RT.

Real-time PCR reaction was performed with a Takara Ex Taq R-PCR kit, version 1.0 (Takara, Otsu, Japan), using a smart cycler system (Cepheid, Sunnyvale, CA, USA). The reaction solution (25 *μ*l) contained Takara Ex Taq HS (5 U *μ*l^−1^), 10 × R-PCR buffer, 250 mM Mg^2+^ solution, 10 mM dNTP mixture, SYBR Green I (1 : 1000 dilution; Cambrex Bio Science Rockland Inc., Rockland, ME, USA) and 20 *μ*M of the primers for EphB4 gene, 2501–2625 in the cDNA (EphB4-S: 5′-ACGGACAGTTCACAGTCATC-3′ and EphB4-AS: 5′-GCAACATCCTAGTCAACAGC-3′) and for ephrinB2 gene, 679–785 in the cDNA (ephrinB2-S: 5′-CAACATCCTCGGTTCCGAAG-3′ and ephrinB2-AS: 5′-CCTCTTGCTGAAGTACCGGA-3′) with the transcribed total RNA from the tissue and a serially diluted standard template. The real-time PCR reactions were initially denatured by heating at 95°C for 30 s, followed by 40 cycles consisting of denaturation at 94°C for 10 s, annealing at 55°C for 5 s and extension at 72°C for 20 s. A strong linear relationship between the threshold cycle and the log concentration of the starting DNA copy number was always shown (correlation coefficient >0.99). Quantitative analysis was performed to determine the copy numbers of each sample.

### Statistical analysis

EphB4 and ephrinB2 mRNA levels were determined from three parts taken from each tumour, and each sample was analysed in triplicate. The differences in the histoscores and mRNA levels of EphB4 and ephrinB2 were analysed by Student's *t*-test. The correlation coefficients were evaluated both by linear regression analysis and bivariate Pearson correlation. Survival rates were calculated using the Kaplan–Meier method and analysed by the log-rank test. Differences were considered significant when *P* was less than 0.05.

## RESULTS

**Figure 1:**
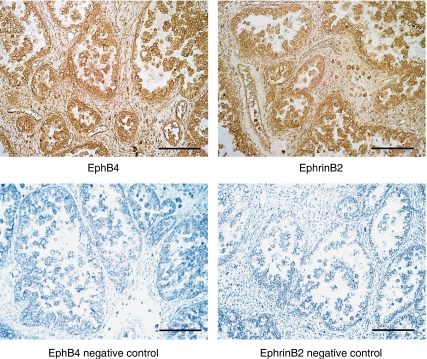
Immunohistochemical staining for EphB4 and ephrinB2 in ovarian cancers (original magnification × 200). A representative case of clear cell carcinoma of the right ovary is shown. Rabbit EphB4 and ephrinB2 antibodies (Santa Cruz) were used at dilutions of 1 : 100 and 1 : 75, respectively, as primary antibodies. Dark brown staining represents positive for EphB4 and ephrinB2 antigen. Bars=100 *μ*m.

**Figure 2:**
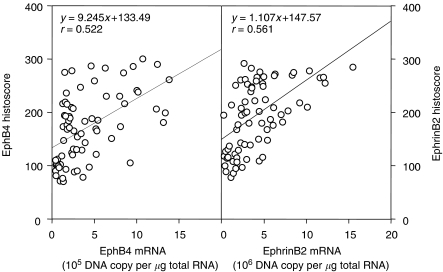
Correlation of EphB4 and ephrinB2 histoscores with mRNA levels in ovarian cancers. Correlation between EphB4 histoscores in cancer cells with mRNA levels and ephrinB2 histoscores in cancer cells with mRNA levels in ovarian cancers is shown. Both EphB4 and ephrinB2 histoscores and mRNA levels were determined by immunohistochemistry and real-time RT–PCR, respectively. Each level is the mean±s.d. of nine determinations.

All the ovarian cancer specimens revealed strong staining for EphB4 and ephrinB2 in the cancer cells and very faint staining in vascular endothelial cells. Positive staining in the cell membrane of the cancer cells has been shown for both EphB4 and ephrinB2. Immunohistochemical staining for EphB4 and ephrinB2 of a representative case of clear cell carcinoma of the right ovary is shown in Figure 1. Both EphB4 and ephrinB2 mRNA seemed to be expressed mainly from the cancer cells. EphB4 as well as ephrinB2 histoscores in cancer cells correlated with the corresponding mRNA levels in each case (EphB4, *y*=9.245*x*+133.49, *r*=0.522, *P*<0.001; ephrinB2, *y*=1.107*x*+147.57, *r*=0.561, *P*<0.001), as shown in Figure 2.

**Figure 3:**
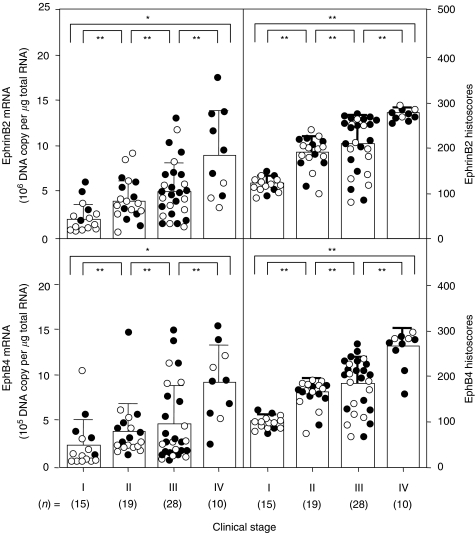
EphB4 and ephrinB2 histoscores and mRNA levels in ovarian cancers classified according to clinical stages. The histoscores and mRNA levels of EphB4 and ephrinB2 were determined by immunohistochemistry and real-time RT–PCR, respectively. Clinical stages of ovarian cancer were assessed according to the FIGO classification. Each level is the mean±s.d. of nine determinations. Alive and dead cases are numbered in open circles and closed circles, respectively; ^*^P<0.05; ^**^P<0.001.

**Figure 4:**
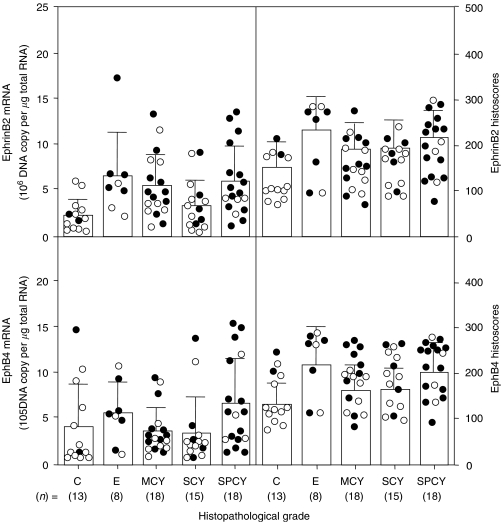
EphB4 and ephrinB2 histoscores and mRNA levels in ovarian cancers classified according to histopathological types. SPCY, serous papillary cystadenocarcinoma; SCY, serous cystadenocarcinoma; MCY, mucinous cystadenocarcinoma; C, clear cell carcinoma; E, endometrioid adenocarcinoma. Alive and dead cases are numbered in open circles and closed circles, respectively. Each level is the mean±s.d. of nine determinations.

Both EphB4 and ephrinB2 histoscores in cancer cells and mRNA levels in ovarian cancers significantly increased according to clinical stage (I<II<III<IV, *P*<0.001), as shown in Figure 3. There was no significant difference in histoscores or mRNA levels of EphB4 and ephrinB2 according to histopathological type, as shown in Figure 4.

We analysed the prognosis of the 72 patients who underwent surgical resection. EphB4 histoscore of 170 and 4.0 × 10^5^ DNA copy per *μ*g total RNA in EphB4 mRNA level and ephrinB2 histoscore of 192 and 3.9 × 10^6^ DNA copy per *μ*g total RNA in ephrinB2 mRNA level were the median values, and were adopted to divide the 72 patients into two groups of 36 patients each.

**Figure 5:**
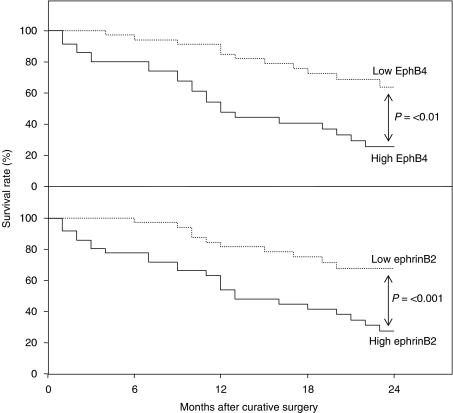
Survival rates after curative resection for ovarian cancers. Patient prognosis was analysed with a 24-month survival rate. High EphB4, histoscores >170 and mRNA levels >4.0 × 10^5^ DNA copy per *μ*g total RNA, *n*=36, solid line; low EphB4, histoscores <170 and mRNA levels <4.0 × 10^5^ DNA copy per *μ*g total RNA, *n*=36, dotted line in the upper panel. High ephrinB2, histoscores >192 and mRNA levels >3.9 × 10^6^ DNA copy per *μ*g total RNA, *n*=36, solid line; low ephrinB2, histoscores <192 and mRNA levels <3.9 × 10^6^ DNA copy per *μ*g total RNA, *n*=36, dotted line in the lower panel.

The 24-month survival rate of the 36 patients with high EphB4 (cases with EphB4 histoscore over 170; the same as those with EphB4 mRNA levels over 4.0 × 10^5^ DNA copy per *μ*g total RNA) was 25%, and the rate of the other 36 patients with low EphB4 (cases with EphB4 histoscore below 170; EphB4 mRNA levels below 4.0 × 10^5^ DNA copy per *μ*g total RNA) was 68%. The survival rate of the 36 patients with high ephrinB2 (cases with ephrinB2 histoscore over 192; the same as those with ephrinB2 mRNA levels over 3.9 × 10^6^ DNA copy per *μ*g total RNA) was 27%, while that of the other 36 patients with low ephrinB2 (cases with ephrinB2 histoscore below 192; ephrinB2 mRNA levels below 3.9 × 10^6^ DNA copy per *μ*g total RNA) was 64%. There was a significant difference (*P*<0.05 in EphB4 and *P*<0.01 in ephrinB2) between the 24-month survival rates of the 36 patients with high or low histoscores and mRNA levels of EphB4 and ephrinB2, as shown in Figure 5.

## DISCUSSION

High levels of Eph expression have been reported in various cancer cell lines and cancer specimens, including cancer of the breast (Zantek *et al*, 1999; Zelinski *et al*, 2001), prostate (Walker-Daniels *et al*, 1999), colon (Rosenberg *et al*, 1997), oesophagus (Miyazaki *et al*, 2003), lung (Kinch *et al*, 2003), uterine endometrium (Alam *et al*, 2007) and in metastatic melanoma (Easty and Benett, 2000). Besides, high levels of Eph expression are found to be associated with more aggressive behaviour in tumours and tumour models, showing tumorigenic and metastatic functions (Rosenberg *et al*, 1997). Increased expression of ephrinB in high-grade ovarian tumours and clear cell and serous carcinomas correlated with tumour aggressiveness, which was associated with higher rates of disease recurrence and poor survival rate (Castellvi *et al*, 2006). Correlated expressions of EphB4 and ephrinB3 with strong expression in epithelial ovarian cancer histotypes address the possibility of involvement of paracrine/juxtacrine signalling through tumour progression (Castellano *et al*, 2006). Elevated expression and activity of Eph receptors have been correlated with the growth of solid tumours (Easty *et al*, 1999). In addition, a high expression of ephrins may be associated with an increased potential for tumour growth, tumorigenicity and metastasis (Kyokawa *et al*, 1994; Vogt *et al*, 1998; Easty *et al*, 1999; Walker-Daniels *et al*, 1999; Tang *et al*, 2000; Miyazaki *et al*, 2003). Among them, EphB4 and ephrinB2 arbitrate the enhanced proliferation, migration and metastatic potential of tumour cells (Vogt *et al*, 1998; Easty *et al*, 1999; Brantley *et al*, 2002; Cheng *et al*, 2002; Lawrenson *et al*, 2002; Pawson, 2002).

EphA2 receptor and its ephrinA1 and ephrinA5 ligands have shown increased expression in ovarian tumours with poor survival rates (Herath *et al*, 2006). In the present study, a significant difference in patient prognoses was found between high and low expression levels of both EphB4 and ephrinB2, which increased during advancement from stage I to stages II, III and IV. Overexpression of EphB4 and ephrinB2 in tumour cells suggests that EphB/ephrinB signalling drives destabilisation, which can also affect cell–matrix attachment, and thereby promote invasion and metastasis (Kyokawa *et al*, 1994).

Accumulating evidence suggests that overexpression or coexpression of Eph family RTKs and their ligands could promote tumour progression. Overexpression of EphB4 and ephrinB2 has been observed in malignant colonic epithelium and uterine endometrial cancer (Sinha *et al*, 2003; Alam *et al*, 2007). EphA2 was found to be expressed at high levels in metastatic melanoma cells in comparison with normal melanocytes (Easty *et al*, 1995). Also, ephrinB2 was highly expressed in primary and metastatic melanomas compared to benign melanocytic nevi (Vogt *et al*, 1998). Furthermore, the expression of ephrinA1 and the upregulation of its receptor, EphA2, were found during the course of melanoma progression (Vogt *et al*, 1998). EphA1 has also been shown to be oncogenic in the classical 3T3 fibroblast assay and coexpression of an ephrin ligand could generate an autocrine loop (Maru *et al*, 1990).

Noren *et al* (2004) proposed a proangiogenic role for EphB4 in tumour progression where the plausible linkage between the EphB4 ectodomain on tumour cells and ephrinB2 in the vasculature seems to facilitate the formation of blood vessels and remodelling (Noren *et al*, 2004). Consistent with this, in our present study, weak staining in vascular endothelial cells, although not very significant, may lead to the hypothesis that there is localised EphB4 activation in the cell membrane of cancer cells that are in contact with blood vessels, which might promote metastasis and further tumour progression. Besides, EphB4 receptor and ephrinB2 ligand were significantly higher and overexpressed with tumour advancement in ovarian cancer regardless of histopathological types. Thus, data from our present study suggest that autocrine stimulation by EphB4 and ephrinB2 coexpression might mediate the advancement of ovarian cancer. The coexpression of EphB4/ephrinB2 may potentiate tumour advancement leading to poor survival and can be recognised as a novel prognostic indicator in the primary tumour of ovarian cancers. In addition, to block the EphB4/ephrinB2 signalling pathway using soluble ephrinB2, an attractive therapeutic strategy might be developed in the future.
